# Supplementary material for: WordCommentsAnalyzer: A windows software tool for qualitative research
Source: F1000Res. 2018 Sep 4;7:536. Originally published 2018 May 3. [Version 2] doi: 10.12688/f1000research.14819.2 (PMC6137411; doi:10.12688/f1000research.14819.2)
Supplement: Supplementary file 3 [file f1000research-7-17614-s0002.tgz › b2a79496-4ada-47c4-9682-a99ce867836d.zip!/Eight Iranian Health Journals/The Journal of Toloo-e-Behdasht_27.docx]

© The Journal of Toloo-e-Behdasht. This work is licensed under CC BY 4.0 (https://creativecommons.org/licenses/by/4.0/).

Volume & Issue: دوره 12 - شماره 4

Title: جذب بیولوژیکی سرب و روی از محلول های آبی سنتتیک بوسیله پودر لجن تصفیه خانه فاضلاب اهواز

http://tbj.ssu.ac.ir/article-1-1710-fa.html

مقدمه: از آنجایی که روشهای موجود جهت جداسازی فلزات سنگین مشکلاتی به همراه دارد‌، اخیراً تمایل به استفاده از روش جذب بیولوژیکی توسط جاذبهای ارزان قیمت رو به گسترش است. در این تحقیق جذب فلزات سنگین سرب و روی توسط پودر لجن خشک شده حاصل از بسترهای لجن خشک کن تصفیه خانه فاضلاب شهری (بیوجاذب) بررسی شده است‌. روش بررسی: آزمایشات بصورت ناپیوسته با دستگاه شیکر در دمای آزمایشگاهی 20 تا 26 درجه سلسیوس و دور اختلاط 200 دور بر دقیقه با تهیه محلولهای 100 میلی لیتری پودر لجن انجام گردیده است‌. پساب مورد بررسی در این پژوهش به صورت سنتتیک با محلول های استاندارد سرب و روی تهیه گردیده است. پارامترهای مورد بررسی عبارت از‌ pH اولیه سیستم از 2 تا 6‌، زمان تماس 5 تا 300 دقیقه‌، غلظت اولیه یون فلز 50‌، 100 و mg/l 150و تعیین زمان تعادلی جذب می باشد. پیش تصفیه بیوجاذب شامل خشک سازی‌، خرد سازی و دانه بندی ذرات بین الک 120 و 230 بوده است‌. یافته ها: بر اساس نتایج بدست آمده از فرآیند بیوجذب سرب و روی‌، حداکثر راندمان جذب یون سرب مربوط به غلظت mg/l 150و به میزان 8/98 درصد و در مدت زمان 5 دقیقه و حداکثر راندمان جذب یون روی مربوط به غلظت mg/l 50 و به میزان 6/35 درصد و در مدت زمان 30 دقیقه بدست آمد. زمان تعادل برای یون سرب 120 دقیقه و برای یون روی 300 دقیقه مشاهده گردید و pH بهینه برای یون سرب و روی به ترتیب 4 و 6 بدست آمد‌. همچنین دوز بهینه بیوجاذب برای یون سرب و روی به ترتیبg/l 2 با حداکثر راندمان حذف 7/98 درصد وg/l 4 با حداکثر 7/72 درصد حذف برآورد شده است‌. ایزوترم جذب فروندلیچ مدل مناسبی برای هر دو فلز بود اما ایزوترم جذب لانگمویر تنها برای فلز روی برازش بسیار دقیقی داشته است و نتایج جذب سرب بسیار دور از آن بود‌. نتیجه گیری: با توجه به نتایج پژوهش حاضر می‌توان گفت‌، پودر لجن دفعی فاضلاب شهری می‌تواند بعنوان جاذبی در دسترس و بسیار عالی برای حذف سرب بکار گرفته شود و همچنین نیز جاذب مناسبی برای حذف روی از پسابهای با غلظت متوسط باشد به شرط آنکه از لجنی استفاده شود که میزان غلظت روی اولیه آن پایین باشد‌.

Title: فرآوری لجن اسیدی تولیدی در صنایع بازیافت روغن موتور مصرف شده با مواد افزودنی به قیر پلیمری

http://tbj.ssu.ac.ir/article-1-1711-fa.html

مقدمه: لجن اسیدی محصول جانبی صنایع فراورش مجدد روغن موتور مصرف شده می باشد که به عنوان ماده زائد خطرناک به محیط زیست دفع می شود. تحت شرایط خاصی از ادغام لجن اسیدی و خاک بنتونیت و پلیمر استایرن بوتادین استایرن( SBS ) قیر پلیمری تولید می شود. هدف این مطالعه بازیافت لجن اسیدی به قیر پلیمری به کمک مواد افزودنی، خاک بنتونیت و پلیمر SBS بود. روش بررسی: در این تحقیق تجربی تاثیر مواد افزودنی با درصدهای وزنی مختلف بر روی پارامترهای کارایی قیر مورد ارزیابی قرار گرفت. در ابتدا فاز روغن هدر رفته به داخل لجن اسیدی با تغلیظ سانتریفیوژی جداسازی شد. سپس لجن اسیدی و مواد افزودنی با میکسر سرعت بالا، 2500 دور در دقیقه در دمای oC180 حدود 30 دقیقه با هم مخلوط شد. در نهایت بر روی لجن اسیدی و فراورده بدست آمده آزمایشات کارایی قیر انجام شد. داده های بدست آمده به کمک برنامه 2007 Excel تجزیه و تحلیل شدند. یافته ها: نتایج نشان دهنده آن است که پارامترهای کارایی شامل دمای نقطه نرمی، افت وزنی، درجه نفوذ، PI، دمای نقطه شکست فراس و حساسیت حرارتی به ترتیب از oC37، 38/1درصد، 230 دهم میلیمتر، 07854/0- ، oC 5- ، 0451/0 به oC54، 1 درصد، 130 دهم میلیمتر ، 7094/2، oC 11- ، 02721/0 ارتقاء یافت. نتیجه گیری: خاک بنتونیت و پلیمر SBS به ترتیب در 2 و4 درصد وزنی به عنوان مواد افزودنی مناسب به منظور بازیافت لجن اسیدی به قیر پلیمری بود

Title: بررسی مقایسه ای مقدار جذب کروم شش ظرفیتی فاضلاب با استفاده از دانه گرانولی و دانه پودر شده اسپند

http://tbj.ssu.ac.ir/article-1-1712-fa.html

مقدمه: کروم شش ظرفیتی یکی از سمی ترین فلزات سنگین بوده که باید قبل از ورود به محیط توسط یک فرایند موثر و کارآمد از پسابهای آلوده حذف شود. هدف از این مطالعه بررسی مقایسه ای مقدار جذب کروم شش ظرفیتی فاضلاب با استفاده از دانه گرانولی و پودر شده اسپند بوده است. روش بررسی: دانه های اسپند بعد از جمع آوری و تمیز سازی الک شده و دانه های با مش 10 به عنوان دانه گرانولی مورد استفاده قرار گرفت. مقداری از دانه های گرانولی توسط آسیاب پودر گردید و سپس ذرات با مش 60 به عنوان دانه پودر شده مورد استفاده قرار گرفت. برای تهیه محلول استوک کروم از دی کرومات پتاسیم استفاده شد و جهت اندازه گیری غلظت کروم از روش رنگ سنجی با استفاده از UV/VIS Spectrometer T80+ استفاده گردید. تمام مواد مورد استفاده محصول شرکت مرک بود. یافته ها: نتایج نشان داد که حداکثر جذب کروم برای هر دو جاذب در pH 5/1 به دست آمد، و با افزایش مقدار جاذب راندمان حذف افزایش یافته ولی ظرفیت جذب کاهش یافت. با افزایش زمان ماند و افزایش سرعت اختلاط مقدار جذب کروم توسط هر دو جاذب روند صعودی داشت اما با افزایش غلظت اولیه کروم این روند سیر نزولی را نشان داد. در تمام آزمایشات مقدار جذب کروم توسط دانه پودر شده اسپند بسیار بیشتر از دانه گرانولی آن به دست آمد. نتیجه گیری: با توجه به نتایج این تحقیق مشخص شد که دانه پودر شده اسپند توانایی بیشتری در جذب کروم نسبت به دانه گرانولی آن دارد. بنابراین دانه پودر شده اسپند می تواند به عنوان یک جاذب ارزان قیمت در حذف کروم مورد استفاده قرار گیرد.

Title: آزمون سمیت فاضلاب رنگی تصفیه شده به روش الکتروفنتون توسط دافنیا مگنا

http://tbj.ssu.ac.ir/article-1-1714-fa.html

مقدمه: آلودگی منابع آب در اثر تخلیه فاضلاب های خروجی صنایع تحدید عمده بر ارگانیسم های زنده محیط زیست آبی است. آزمایش های فیزیکی و شیمیایی برای ارزیابی اثرات بالقوه این آلاینده ها بر حیات آبزیان کافی نیست .استفاده از آزمون های زیستی می تواند معیار مناسب و مستقیمی از سمیت را برای تکمیل معیار های فیزیکوشیمیایی کیفیت فاضلاب ها فراهم نماید. روش بررسی: دافنیای مورد نیاز از مرکز تکثیر و پرورش ماهی اصفهان تهیه و پس از آماده سازی غلظت های مختلف نمونه های فاضلاب خام و نمونه های رنگزدایی شده در هشت درصد حجمی مختلف، به تمام ظروف حاوی نمونه فاضلاب خام و رنگ زدایی شده و نمونه شاهد تعداد 10 عدد نوزاد دافنیا که در آب رقیق سازی شستشو شده اند، اضافه گردید. مشاهده نمونه ها بعد از 48 ساعت انجام و تعداد حیوانات مرده در هر ظرف آزمایش شمارش و ثبت گردید. در پایان آزمایش ها، واحد سمیت حاد، غلظت کشنده 48 ساعته و بازده حذف سمیت در شرایط بهینه فرآیند الکتروفنتون محاسبه گردید. یافته ها: بر اساس نتایج بدست آمده از آزمون سمیت مشخص گردید که h48 LC50 – در نمونه های فاضلاب سنتتیک با غلظت رنگ mg/L 40 از 18 درصد حجمی قبل از حذف رنگ به 70 درصد حجمی بعد از تصفیه، نمونه های فاضلاب سنتتیک با غلظت رنگ mg/L 100 از 14 به 50 درصد، فاضلاب سنتتیک با غلظت رنگ mg/L 200از 10 به 35 درصد و در نمونه های حاوی رنگ با غلظت mg/L 300 از 8/6 به 20 درصد حجمی افزایش پیدا کرد. همچنین بازده حذف سمیت برای غلظت های مختلف رنگ نیز به ترتیب 76، 72 ،71 و 66 درصد بدست آمد. نتیجه گیری: با توجه به نتایج بدست آمده می توان اظهار داشت که زیست آزمونی جهت ارزیابی پتانسیل سمیت فاضلاب های رنگی جهت تخلیه آنها به درون آبهای سطحی وتعیین معیار های کیفیت جهت تعیین غلظت های مجاز این آلاینده ها روشی ساده، سریع وموثر می باشد.

Title: جذب کروم از محلول های آبی توسط سرباره صنایع فولاد: مدل سازی ایزوترم و سینتیک جذب

http://tbj.ssu.ac.ir/article-1-1715-fa.html

مقدمه: یکی از عمده ترین آلاینده های محیط زیست پساب های صنعتی حاوی فلزات سنگین است. کروم از جمله فلزات سنگین می باشد که در پساب صنایع مختلف وجود دارد و به شدت برای انسان و محیط زیست سمی و خطرناک است. سرباره فولاد یک محصول جانبی در صنایع فولاد است که به میزان قابل توجهی تولید می گردد. هدف از این تحقیق بررسی حذف فلزات سنگین مثل کروم از محیط های آبی با استفاده از سرباره صنایع فولاد به عنوان جاذب ارزان قیمت بود. روش بررسی: در این مطالعه آزمایشگاهی حذف کروم با بکارگیری سرباره حاصل از صنایع فولاد با تغییر پارامترهایی از جمله pH ، زمان تماس، مقدار ماده جاذب(سرباره) و غلظت اولیه کروم در سیستم ناپیوسته مورد بررسی قرار گرفت. نمونه پساب مصنوعی مورد استفاده برای انجام آزمایشات برای ساخت محلول کروم با اضافه کردن دی کرومات پتاسیم در آب مقطر و ساخت غلظت های25، 20، 15، 10، 5 میلی گرم بر لیترآماده گردید. یافته ها: نتایج این تحقیق نشان می دهد که در شرایط بهینه سرباره قابلیت جذب یون های کروم از محلول های آبی با غلظت 25 میلی گرم بر لیتر،7pH= ، مقدار جاذب 3 گرم و زمان تماس 60 دقیقه را تا حدود 3/96 درصد داراست. داده های آزمایش از مدل های لانگمویر و سینتیک جذب شبه مرتبه دوم تبعیت کردند. حداکثر ظرفیت جذب (qm) با مدل لانگمویر معادل 97/. میلی گرم برگرم بود. و ضریب همبستگی (R2 ) در سینتیک جذب شبه مرتبه دوم معادل 1 می باشد. خصوصیات فیزیکی، شیمیایی و مورفولوژی سرباره نیز با استفاده از دستگاه های XRD,XRF,SEM تعیین گردید. نتیجه گیری: نتایج حاصل از این تحقیق می تواند در به کارگیری سرباره صنایع فولاد جهت حذف کروم از محلول های آبی مفید باشد.

Title: بررسی اثر منعقدکننده ها در حذف CODوTSS شیرابه زباله کارخانه کمپوست اصفهان

http://tbj.ssu.ac.ir/article-1-1716-fa.html

مقدمه: در بسیاری از کشور های جهان شیرابه زباله بدون هیچ تصفیه ای در محیط رها می شودو به دلیل ترکیب متفاوت شیرابه زباله در مکان های مختلف ، تاکنون روش یکنواختی برای تصفیه آن ارائه نشده است . عدم کنترل و بی توجهی نسبت به تصفیه و دفع صحیح شیرابه موجب آلودگی محیط می گردد. این تحقیق با هدف بررسی اثرمنعقدکننده ها در حذف CODوTSS شیرابه زباله کارخانه کمپوست اصفهان انجام شد. بدین منظور از مواد منعقدکننده سولفات آهن،کلرور آهن،پلی فریک سولفات، آلوم و پلی آلومینیوم کلراید به همراه دو کمک منعقد کننده تجاری آنیونی و کاتیونی استفاده گردید. روش بررسی: در این مطالعه که از نوع تجربی- آزمایشگاهی می باشد، نمونه شیرابه از حوضچه جمع آوری و ذخیره شیرابه کارخانه کمپوست اصفهان برداشت شد. در ابتدا خصوصیات شیرابه بر اساس روش استاندارد اندازه گیری گردید. در مراحل بعد بااستفاده از روش آزمایش جار اثر تغییرات دوز مواد منعقد کننده (5/0، 1، 5/1 ،2 ، 5/2 و3 گرم بر لیتر) وpH (12، 11 ، 9-4) در حذف CODو TSS بررسی گردید و نهایتا دوز موثر، pH بهینه و بهترین منعقد کننده مشخص شد. یافته ها: حداکثر راندمان حذف TSSو CODبا استفاده از پلی فریک سولفات بدست آمدکه در دوز g/l2پلی فریک سولفات در 11 pH= ،49 درصد حذف COD و دردوزg/l 5/2 ، 51 درصدحذف TSS بدست آمد.کمک منعقد کننده K350CF به همراه منعقد کننده پلی فریک سولفات بیشترین افزایش راندمان حذف CODوTSS را داشته و به ترتیب برابر 53 و 52 درصد می باشد. بیشترین راندمان حذفCOD در حضور کمک منعقد کننده LT25را پلی آلومینیوم کلراید داشته که برابر 49درصد بود. نتیجه گیری: در حذف CODو TSS،موثرترین منعقدکننده پلی فریک سولفات بوده که در pHبازی نتیجه بهتری را نشان می دهد. با توجه به نتایج بدست آمده می‌توان روش انعقاد وکواگولاسیون را روشی مناسب و ارزان قیمت جهت پیش تصفیه شیرابه حاصل از کارخانه کمپوست اصفهان و آماده سازی آن برای تصفیه بهتر و مناسب تر باروشهای دیگر معرفی نمود.

Title: کاربرد خاکستر استخوان به عنوان جاذب طبیعی درحذف رنگ اسیدی نارنجی 7 از فاضلاب سنتتیک نساجی

http://tbj.ssu.ac.ir/article-1-1717-fa.html

مقدمه: صنایع نساجی فاضلاب رنگی با ویژگی‌های متفاوتی تولید می‌کنند که باید تصفیه شوند. فرآیند جذب سطحی از فرآیند‌های کارآمد در حذف رنگ می‌باشد. هدف از این مطالعه کاربرد خاکستر استخوان به عنوان جاذب طبیعی برای حذف رنگ اسیدی نارنجی 7 از فاضلاب سنتتیک نساجی می‌باشد. روش بررسی: محلول رنگ اسیدی نارنجی 7 تهیه و اثر متغیرهای زمان تماس، pH، غلظت اولیه رنگ، دوز جاذب و شدت اختلاط بر روی کارایی حذف رنگ مورد بررسی قرار گرفت. سنجش غلظت رنگ در نمونه‌ها به روش اسپکتروفتومتری در طول موج 484 نانومتر انجام شد. یافته‌ها: نتایج نشان داد pH نقش مهمی در جذب رنگ داشته است، به طوری که با کاهش pH، میزان جذب رنگ در واحد جرم جاذب افزایش یافته است. حداکثر ظرفیت جذب سطحی رنگ بر روی خاکستر استخوان 74/0 میلی‌گرم بر گرم در 3pH: اتفاق افتاده است. نتایج نشان داد که با کاهش غلظت اولیه رنگ و با افزایش دوز جاذب، مقدار آلاینده جذب شده در واحد جرم جاذب کاهش می‌یابد. با افزایش دوز جاذب از 5/0 به 4 گرم در 100 میلی‌لیتر راندمان حذف رنگ افزایش می یابد. با کاهش غلظت اولیه رنگ از 100 به 25 میلی‌گرم در لیتر در 3pH: و زمان 180 دقیقه راندمان حذف از10% به 96% درصد افزایش یافته است. در غلظت 25 و 50 میلی‌گرم در لیتر با افزایش شدت اختلاط از 40 به120 دور در دقیقه، میزان ظرفیت جذب رنگ در واحد جرم جاذب افزایش یافته است. همچنین مشخص گردید که زمان مناسب جهت دستیابی به حداکثر جذب رنگ و حالت تعادل، 180 دقیقه می‌باشد. نتیجه‌گیری: به دلیل سهولت تهیه، فراوانی مواد اولیه و عملکرد قابل توجه خاکستر استخوان به عنوان جاذب طبیعی در حذف آلاینده‌ها از فاضلاب نساجی، این جاذب جهت حذف رنگ موثر می‌باشد

Title: کارایی هیبرید نانولوله های کربنی چند جداره اصلاح شده با هیپوکلریت کلسیم و نانوذرات سیلیکا در حذف بنزن از محلول های آبی

http://tbj.ssu.ac.ir/article-1-1718-fa.html

مقدمه: بنزن یک ترکیب آلی فرار در محصولات نفتی است،که مهمترین نگرانی در مورد آن خاصیت سرطانزایی بنزن می باشد.. از این روهدف از این مطالعه بررسی قابلیت حذف بنزن با استفاده ازنا نو لوله های کربنی چند دیواره اصلاح شده با هیپوکلریت کلسیم، نانوذرات سیلیکا و هیبرید نانولوله های کربنی چند دیواره اصلاح شده با هیپوکلریت کلسیم از محلول های آبی است. روش بررسی: مطالعه حذف بنزن یک مطالعه تجربی-آزمایشگاهی است. دراین مطالعه غلظت بنزن mg/l100، غلظت نانولوله‌های کربنی اصلاح شده و نانو ذرات سیلیکا و هیبرید نانولوله های کربنی چند دیواره اصلاح شده با هیپوکلریت کلسیم و نانو ذرات سیلیکا g/l5/0 و زمان تماس 1ساعت و 2ساعت وpH خنثی برای انجام آزمایش در نظر گرفته شد. نمونه‌های مورد نیاز به صورت سنتتیک در آزمایشگاه ساخته شد و توسط دستگاه گاز کروماتوگرافی جرمی آنالیز گردید. یافته‌ها: نتایج نشان می‌دهند که بهترین ظرفیت جذب(qe) و راندمان حذف (R) برای بنزن، توسط هیبرید نانولوله‌های کربنی چند دیواره اصلاح شده باهیپوکلریت کلسیم و نانوذرات سیلیکا mg/g)46/192 qe=و 23/96%=R) بوده است. همچنین نانولوله‌های کربنی چند دیواره اصلاح شده با هیپوکلریت کلسیم mg/g)58/188qe= و 92/94%=R) و نانوذرات سیلیکا (mg/g6/182qe= و 3/91%=R) می‌باشد. نتیجه گیری: هیبرید نانولوله های کربنی اصلاح شده با هیپوکلریت کلسیم می تواند به طور مؤثری بنزن را از آب جذب نماید و دارای پتانسیل خوبی برای تصفیه آب وفاضلابهای آلوده به مواد نفتی است

Title: بررسی شاخص های بهداشت محیطی هتل ها ، هتل آپارتمان ها و میهمان پذیر های شهر شیراز

http://tbj.ssu.ac.ir/article-1-1719-fa.html

چکیده مقدمه: بسیاری از کشورها صنعت گردشگری را به عنوان منبع اصلی درآمد می دانند. این تحقیق با هدف تعیین شاخص های بهداشت محیطی مراکز اقامتی شهر شیراز و نقش مشاور بهداشتی و مدرک دوره هتل داری متصدی مراکز اقامتی در بالا بردن میزان شاخص های بهداشتی مراکز اقامتی شهر شیراز، درسال 1389انجام گردید. روش بررسی: این مطالعه، به صورت توصیفی_مقطعی انجام شد. برای تعیین شاخص های بهداشت محیطی اماکن با توجه به آئین نامه ماده 13، چک لیستی شامل 52پرسش برای میهمان پذیر هاو83 پرسش برای هتل ها و هتل آپارتمان ها تهیه شد. سپس از طریق مصاحبه حضوری و بازدید از کلیه اماکن اقامتی شهر شیراز شامل 141مکان، تکمیل گردید. داده ها با استفاده از نرم افزار SPSS تجزیه و تحلیل گردید. یافته ها: نتایج نشان داد که در شیراز 2/58 درصد اماکن دارای شرایط بهداشتی،9/31 درصد دارای معیار بهسازی و9/9 درصد در معیار نامطلوب قرار دارند. نتیجه گیری: با الزام کردن هتل ها به داشتن مشاور بهداشتی می توان شاخص های بهداشت محیطی را در هتل ها بالا برد. از طرفی با ملزم کردن داشتن مدرک هتل داری مدیر مراکز اقامتی می توان شاخص های بهداشتی را در مراکز اقامتی ارتقاء داد.

Title: حذف سیانید از محلول های آبی با استفاده ازرزین نانو ذرات آهن (لیواتیت FO36)

http://tbj.ssu.ac.ir/article-1-1721-fa.html

چکیده مقدمه: سیانید یکی از آلاینده های محیط زیست است که در پساب صنایع مختلف وجود دارد و موجب آلودگی منابع آب شده که برای انسان و محیط زیست خطرناک می باشد. سیانید را می‌توان به روش های مختلفی از محیط‌های آبی حذف کرد، اما غالب این روش‌ها هزینه ‌بر می‌باشند. در این مطالعه از رزین تبادل‌کننده لیواتیت FO36 برای حذف سیانید در سیستم ناپیوسته استفاده شده است. روش بررسی: حذف سیانید از محلول های آبی با رزین تبادل‌کننده لیواتیت FO36 در وضعیت‌های مختلف از جمله اثر میزان ماده جاذب، غلظت سیانید اولیه، زمان تماس و pH در فاز ناپیوسته مورد بررسی قرار گرفت. یافته ها: در پروسه تبادل‌کننده ناپیوسته، زمان تعادل بعد از 90 دقیقه تماس حاصل گردید و میزان حداکثر حذف سیانید، در میزان pH 5 تا 9 برای غلظت سیانید، 1 میلی‌گرم در لیتر، به‌دست آمد. لیواتیت FO36 قدرت باند شدن بالایی دارد. ثابت تعادل که به ظرفیت تبادل یون بستگی دارد توسط ایزوترم جذب لانگمیر به‌دست آورده شد. میزان حداکثر ظرفیت جذب سیانید برای FO36 در 5/6= pH به‌دست آمد و اثر تبادل‌کنندگی سیانید بر روی رزین تبادل‌کننده کاتیونی از معادله واکنش درجه اول پیروی کرد. نتیجه گیری: با توجه به یافته های این تحقیق. رزین لیواتیت FO36 می‌تواند به‌عنوان یک گزینه مناسب با توجه به قابل احیاء بودن آن، جهت کاهش میزان سیانید از محیط‌های آبی به‌کار رود

Title: بررسی نگرش ها و رفتار های اجتماعی مردم شهر یزد نسبت به مصرف داروهای گیاهی در درمان دیابت

http://tbj.ssu.ac.ir/article-1-1722-fa.html

مقدمه:دیابت شایع ترین بیماری متابولیک در جهان است .قبل از کشف انسولین و داروهای کاهش دهنده قندخون، بیماران دیابتی با گیاهان دارویی و درمان های سنتی معالجه می‌شدند. هم اکنون داروهای متعددی جهت کاهش میزان قند خون بالا ارایه شده است ولی به دلیل عدم بهبودی کامل این بیماری با مصرف داروهای موجود، تمایل به استفاده از درمان‌های جایگزین و سنتی افزایش یافته است.به همین منظور شایسته است جهت شناساندن فواید وعوارض گیاهان دارویی تلاش بیشتری صورت گیرد لذا این مطالعه با هدف بررسی نگرش هاو رفتار های اجتماعی مردم شهر یزد نسبت به مصرف داروهای گیاهی در درمان دیابت انجام شد. روش بررسی: این مطالعه ازنوع توصیفی می‌باشدکه به روش مقطعی Cross-sectional درسال 1391انجام گردیده است. در این مطالعه 300 فرد 15 تا 65 سال واجد شرایط به شیوه تصادفی از 10 نقطه شهر یزد انتخاب شدند.اطلاعات ازطریق پرسشنامه جمع آوری و با آزمون های آماری مورد تجزیه و تحلیل قرار گرفت. نتایج: نمره نگرش مرد و زن در مورد مصرف داروهای گیاهی دردرمان دیابت یکسان است وهردو گروه جنسی دارای اعتقادخوبی نسبت به مصرف گیاهان دارویی بودند. هرچه سن بالاتر می‌رود نگرش آنها به مصرف گیاهان دارویی افزایش می‌یابد که با 020/P = معنی دار گزارش شد. بررسی نگرش افراد برحسب سطح تحصیلات، با 026/P = معنی دارگزارش شد,بدین معنی که با افزایش تحصیلات Attitude کاهش می‌یابد، که پیشنهاد می‌شود مسئولین مربوطه از جمله رسانه ها و آموزش عالی به این مساله توجه بیشتری داشته باشند. نتیجه گیری: مشخص شد افردی که دیابت داشتند آگاهی بیشتری در مورد نوع گیاه دارویی کاهنده قند خون دارند.وافراد اطلاعات خود را بیشتر از اقوام وبستگان درجه یک کسب کرده بودند

Title: بررسی کارآیی سیستم بیوفیلمی با بستر مستغرق (DAUOSBR) در حذف مواد آلی(BOD5 و COD)، کدورت و کل جامدات معلق از فاضلاب بیمارستانی

http://tbj.ssu.ac.ir/article-1-1723-fa.html

چکیده مقدمه: امروزه فرآیندهای تصفیه هوازی با رشد چسبیدۀ ثابت، به دلیل جداسازی مواد آلی در فاضلاب و نیز به منظور نیتریفیکاسیون(تبدیل آمونیوم به نیترات)، توجه مهندسین و طراحان تصفیه خانه های فاضلاب را به خود جلب کرده است. هدف از این مطالعه بررسی کارایی سیستم بیوفیلمی با بستر مستغرق در حذف مواد آلی(BOD5 و COD)، کدورت و جامدات معلق(TSS)از فاضلاب بیمارستان فارابی کرمانشاه می باشد. روش بررسی: در این مطالعه ابتدا اقدام به ساخت راکتور، راه اندازی و بارگذاری سیستم لجن فعال رشد ثابت با بستر مستغرق شد و سپس جهت بررسی راندمان سیستم در حذف مواد آلی(BOD5 و COD)، کدورت و جامدات معلق(TSS) در سه زمان ماند مختلف(9/2ساعت هوازی و 1/1 ساعت بی هوازی، 6/3ساعت هوازی و 4/1 ساعت بی هوازی و 4ساعت هوازی و 5/1 ساعت بی هوازی)اقدام به برداشت 135 نمونه شد. یافته ها:نتایج نشان داد که میزان حذف COD،BOD5،TSS و کدورت در مرحله سوم بارگذاری(4ساعت هوازی و 5/1 ساعت بیهوازی) بالاترین میزان را به خود اختصاص داد. به طوری که میانگین حذف پارامترهای مذکور به ترتیب 82 ،95،4/98 و 98 درصد به دست آمد. هرچند که مقایسه میانگین راندمان حذف پارامترهای مذکور(به جز کدورت وBOD) اختلاف معناداری را نشان نداد. نتیجه گیری:در صورت بهره برداری و نگهداری مناسب، سیستم بیوفیلمی با بستر مستغرق کارایی بالایی در حذف مواد آلی، جامدات معلق و کدورت فاضلاب بیمارستانی دارد، بدون اینکه نیازی به برگشت جامدات بیولوژیکی داشته باشد.

Title: ارزیابی اثر pH محلول و غلظت جاذب نانو لوله کربنی چند دیواره اصلاح شده بر کارایی حذف رنگ اریکروم بلک تی از پساب کارخانه سامان نوش همدان

http://tbj.ssu.ac.ir/article-1-1724-fa.html

مقدمه: رنگ‌ها یکی از آلاینده‌های مهم در پساب‌های صنعتی محسوب می‌شوند که تصفیه آن ها به دلیل منشاء مصنوعی و ماهیت مولکولی پیچیده بسیار دشوار است. رنگ اریکروم بلک تی یکی از رنگ‌های خانواده آزو می‌باشد که به صورت گسترده‌ای در مواد غذایی، صنعتی، آرایشی، بهداشتی و دارویی مورد استفاده قرار گرفته است و با توجه به حلالیت بالا در محیط های آبی، زمان ماندگاری بالا، تجزیه ناپذیری توسط ترکیبات ارگانیک و تجمع زیستی آن، در محیط زیست به عنوان یک آلاینده مهم محیط های آبی، به خصوص آب های پذیرنده مطرح است. لذا این مطالعه با هدف بررسی کارایی فرآیند حذف رنگ اریکروم بلک تی توسط نانو لوله کربنی چند دیواره اصلاح شده از نمونه حقیقی پساب صنعتی کارخانه سامان نوش همدان انجام شد. روش بررسی: در این پژوهش به منظور ارزیابی کارایی حذف رنگ اریکروم بلک تی تحت شرایط بهینه از پساب کارخانه سامان نوش همدان به عنوان نمونه حقیقی، از نانو لوله های کربنی چند دیواره اصلاح شده و روش فرآوری Batch استفاده و بدین منظور تاثیر پیراسنجه های pH محلول و غلظت جاذب، مورد ارزیابی قرار گرفت. همچنین به منظور تعیین ثابت سرعت فرآیند جذب، داده های آزمایشگاهی با دو مدل سینتیکی شبه مرتبه اول و دوم مقایسه و با ایزوترم های لانگمویر و فروندلیخ مطابقت داده شدند. نتیجه گیری: نتایج بیانگر آن بود که در شرایط بهینه پیراسنجه ها (3pH= ، مقدار 01/0 گرم جاذب)، کارایی حذف رنگ به 98 درصد رسید. همچنین مشخص شد که داده ها از سینتیک مرتبه دوم و ایزوترم جذب فروندلیخ با ضریب همبستگی 9622/0 پیروی می کنند

Title: مقایسه روش بیولوژیکی و بیوالکتروشیمیایی در تصفیه فاضلابهای حاوی فرمالدوئید

http://tbj.ssu.ac.ir/article-1-1725-fa.html

چکیده مقدمه: رشد سریع صنعت و تکنولوژی در چند سده اخیر و افزایش استانداردهای زندگی باعث شده است که تحقیقات مختلفی جهت یافتن روشهای اقتصادی و کارآمدترتصفیه فاضلاب انجام پذیرد.اگرچه تحقیقات زیادی بر روی دو روش بیولوژیکی و الکتروشیمیایی در حذف آلاینده های سمی صورت پذیرفته است، لیکن ترکیب این دو روش تاکنون مورد بررسی دقیق قرار نگرفته است. هدف از این مطالعه مقایسه مابین راندمان راکتور رشد چسبیده مستغرق و راکتور بیوالکتروشیمیایی (ترکیبی از روش الکتروشیمیایی و راکتور رشد چسبیده مستغرق) بود. روش بررسی: راکتورهای بیولوژیک و بیوالکتروشیمیایی به صورت جداگانه برای تصفیه فاضلاب آلوده به فرمالدوئید برای مدتی طولانی مورد پایش قرار گرفتند. جهت بررسی تاثیر عبور جریان الکتریکی بر روی میکروارگانیسم ها و راندمان سیستم بیولوژیکی از الکترودهای گرافیتی استفاده گردید و راندمان سیستم بطور مداوم مورد پایش قرار گرفت. یافته ها: نتایج این مطالعه نشان داد که راکتور بیولوژیکی رشد چسبیده مستغرق، قادر به حذف 88% فرمالدوئید ورودی به سیستم بود. لیکن با نصب الکترود ها و عبور جریان برق از آن راندمان آن به 85% کاهش یافت. غلظت میکروارگانیسم ها در راکتور بیوالکتروشیمیایی نیز در مقایسه با راکتور بیولوژیک به مراتب کمتر بود. نتایج این تحقیق نشان داد که جریان الکتریکی عبوری از راکتور الکتروبیوشیمیایی رابطه ای معکوس با راندمان حذف فرمالدوئید دارد. نتیجه گیری: نتایج این مطالعه نشان داد که راکتور بیوالکتروشیمیایی و بیولوژیکی در ولتاژ های پایین توانایی تقریباً یکسانی در حذف فرمالدوئید از فاضلاب دارند، با این حال مقدار انرژی مورد نیاز راکتور بیوالکتروشیمیایی بیش از مقدار مورد نیاز راکتور بیولوژیکی است. در ولتاژهای بالا نیز توانایی این راکتور در حذف آلاینده ها به شدت کاهش می یابد. لذا در این مطالعه نتیجه گیری شد که با دانش کنونی روشهای بیوالکتروشیمیایی مقرون به صرفه نیستند

Title: بررسی شیوع آفلاتوکسین در نمونه‌های پسته فرآوری شده شهرستان رفسنجان طی سال‎های90 تا91 و ارتباط آن با زمان برداشت

http://tbj.ssu.ac.ir/article-1-1726-fa.html

چکیده مقدمه: آلودگی میوه پسته به سم آفلاتوکسین ناشی از گونه‌های قارچ Aspergillus یکی از مهمترین مشکلات بهداشتی در مصرف این محصول با ارزش غذایی است. یکی از روش‌های مهم کاهش آفلاتوکسین در توده پسته، غربال یا جداسازی میوه های پسته آلوده است. در این تحقیق پراکندگی آفلاتوکسین در پسته‌های فرآوری شده یک ترمینال در 3 زمان دهم شهریور، بیست و پنجم شهریور و دهم مهر ماه مورد بررسی قرار گرفت. روش بررسی: نمونه‌برداری از پسته‌های خشک جمع‌آوری شده از فاز زیرآبی که شامل پسته‌های نهایی، ریز و با رنگ زرد، و از فاز روآبی چرخ انجام شد. پسته‌های نهایی و ریز به لکه دار، بدشکل و بدون‌لکه، و پسته‌های با رنگ زرد و روآبی به لکه‌دار، بدشکل و با رنگ زرد تقسیم بندی شدند.آفلاتوکسین در نمونه‌ها با استفاده از روش کروماتوگرافی مایع با کارایی بالا (HPLC) اندازه‌گیری گردید. یافته‌ها: از نظر کلی و با استفاده از آزمون‌های آماری مورد استفاده (05/0=p) جهت آنالیز داده‌ها، حاکی از وجود تفاوت در بین انواع پسته‌های مورد بررسی از نظر غلظت آفلاتوکسین‌ها می‌باشد. نتایج نشان داد که وجود پسته‌های بدشکل و لکه دار در آلودگی یک توده پسته ارائه شده به بازار نقش مهمی دارند و دامنه غلظت آفلاتوکسین 1B در آنها به ترتیب از 76/0 تا ng/g 6/251 و 46/1 تا ng/g 3/85در سه زمان نمونه‌برداری متغیر بود. در خصوص پسته‌های ریز مقدار آفلاتوکسین از 23/1 تا 55/241 ng/g متغیر بود، که بسته به بدشکل بودن و وجود و یا عدم وجود لکه بر روی پوست استخوانی متغیر بود. از طرف دیگر عدم وجود لکه بر روی پوست استخوانی در پسته‌های ریز نمی‌تواند دلیلی بر عدم وجود آفلاتوکسین باشد. پسته‌های زرد رنگ نیز یکی از منابع اصلی آلودگی دریک توده پسته بودند و بیشترین غلظت آفلاتوکسین (ng/g 5214) مربوط به آنها بود. مقایسه زمان‌های مختلف نمونه‌برداری نشان داد که بیشترین مقدارآفلاتوکسین با زمان 10 مهر ارتباط دارد. با تاخیر در زمان برداشت، مقدار آفلاتوکسین بین 2 تا 30 برابر در انواع پسته‌های مورد بررسی افزایش یافت. نتیجه گیری: نتایج این تحقیق می‌تواند در جداسازی پسته‌های آلوده از سالم در مرحله فرآوری و یا پس از آن در یک توده پسته مورد استفاده قرار گیرد

Title: بررسی تاثیر عمق استخر و جنسیت شناگران بر شش شاخص کیفیت میکروبی در استخر شنای ازن زنی شده شهر دامغان

http://tbj.ssu.ac.ir/article-1-1727-fa.html

چکیده مقدمه: کاربرد ازن در استخرهای شنا در ایران رو به افزایش است. هدف این مطالعه توصیفی – مقطعی، تعیین کارآیی ازناسیون و تاثیر عمق استخر و جنسیت شناگران بر شش شاخص کیفیت میکروبی بوده است. روش بررسی: در این پژوهش مجموع کلیفرم، اشرشیاکلی، باکتریهای هترتروف، استافیلوکوکوس اورئوس، استرپتوکوک مدفوعی و سودوموناس آئروژینوزا مورد بررسی قرارگرفتند. نمونه برداری در دو نوبت آقایان و بانوان و از هر سه منطقه کم عمق، متوسط و عمیق و در زمان حضور پرتعداد شناگران انجام شده است. یافته ها: براساس نتایج، تعداد باکتریهای هترتروف، اشرشیاکلی و سودوموناس آئروژینوزا همواره بیش از حد مجاز بوده است. آلودگی به استافیلوکوکوس اورئوس در تمام نمونه ها ی بخش آقایان کمتر از حد استاندارد و در10 درصد از نمونه های بخش بانوان بیش از حد مجاز و آلودگی به استرپتوکوک مدفوعی در تمام نمونه ها کمتر از حد مجاز بوده‌است. نتیجه‌گیری: در بسیاری از موارد، تعداد شاخصها از حد استاندارد بیشتر بوده است که می‌تواند ناشی از عواملی همچون عدم کفایت غلظت ازن زنی و یا نامناسب بودن نقاط ازن زنی باشد. همچنین در اغلب موارد، میانگین آلودگی میکروبی در نوبت بانوان بیش از نوبت آقایان بوده‌است که اگر چه اختلاف بین آنها در حد 05/0=α معنی دار نبوده‌ است اما لزوم توجه بیشتر به استخرهای شنا در نوبت بانوان را نشان می دهد

Title: مطالعه عملکرد منیزیم با ظرفیت صفر در احیاء کروم از محلول های آبی

http://tbj.ssu.ac.ir/article-1-1728-fa.html

چکیده مقدمه: کروم از جمله فلزات سنگینی است که به دلیل فراوانی استفاده در مصارف مختلف و عدم تصفیه مناسب فاضلاب صنایع به سهولت وارد منابع آبی شده و منجر به آلودگی وسیع آنها می شود. از این رو در این مطالعه احیاء شیمیایی کروم شش ظرفیتی توسط گرانول منیزیم و تاثیر پارامترهای مختلف بر عملکرد آن مورد بررسی قرار گرفته است. روش بررسی: پژوهش حاضر یک مطالعه تجربی آزمایشگاهی بوده که از فلز منیزیم با ظرفیت صفر به منظور احیاء کروم شش ظرفیتی استفاده شده است. نمونه های مائی حاوی کروم شش ظرفیتی به صورت سنتتیک با استفاده از آب مقطر تهیه گردید. مراحل مختلف انجام آزمایشات در ارلن های 250 میلی لیتری شیشه ای در دستگاه شیکر انکوباتور انجام گردیده و اثر فاکتورهای مختلف نظیر تغییرات pH محلول، غلظت های مختلف منیزیم، غلظت اولیه کروم، دما، زمان تماس و عوامل مداخله کننده مورد بررسی قرار گرفت. در خاتمه غلظت کروم در زمان های مختلف با استفاده از دستگاه اسپکترفوتومتر و در طول موج 540 نانومتر قرائت گردید. یافته ها: نتایج مطالعه اخیر حاکی از آنست که راندمان حذف کروم با استفاده از منیزیم صفر ظرفیتی با افزایش غلظت منیزیم، افزایش غلظت اولیه کروم و دما افزایش و با افزایش pH محلول کاهش داشته است. بیشترین کارایی حذف در زمان 10 دقیقه از انجام واکنش در3=pH، غلظت منیزیم 7/0 گرم در لیتر و کروم اولیه 150 میلی گرم در لیتر به میزان 2/92% بوده است. همچنین وجود اسید هیومیک، سختی و قلیائیت به عنوان عوامل مداخله کننده سبب کاهش کارایی حذف کروم در این فرایند شده است. با افزایش زمان تماس نیز راندمان حذف افزایش یافته است. نتیجه گیری: فلز منیزیم بدلیل خصوصیات منحصر به فرد از جمله بالا بودن پتانسیل اکسیداسیون و احیاء، سهولت دسترسی و بالا بودن استاندارد یون منیزیم در آب قادر است به نحو چشمگیری در حذف کروم موثر بوده و در فرایند حذف کروم در محلول های مائی مورد استفاده قرار گیرد

Title: بررسی کارایی نانولوله های کربنی چند جداره در حذف رنگ راکتیو قرمز 198 از محیط های آبی

http://tbj.ssu.ac.ir/article-1-1729-fa.html

چکیده مقدمه: رنگها از جمله مهمترین آلاینده های موجود در فاضلاب صنایع نساجی و رنگرزی هستند.این ترکیبات غالباً سمی‌‌، جهش زا و غیرقابل تجزیه بیولوژیک می باشند. رنگزاهای آزو به عنوان دسته ای از رنگزاها، کاربردهای گوناگونی در صنایع مختلف داشته که پتانسیل جهش زایی و سرطان زایی برخی از این نوع رنگزاها مشخص شده است. رنگ راکتیو قرمز 198 یکی از رنگ های آزو بوده که در حال حاضر نیز در صنایع نساجی کاربرد دارد. این مطالعه با هدف بررسی کارایی نانولوله های کربنی چند جداره در حذف رنگ راکتیو قرمز 198 از محیط های آبی و نیز تعیین ایزوترم های جذب انجام پذیرفت. روش بررسی: مطالعه حاضر به صورت تجربی و در مقیاس آزمایشگاهی در یک سیستم بسته انجام پذیرفت. در این تحقیق اثر غلظت اولیه رنگ (200-100-50-10 میلی گرم بر لیتر)، جرم جاذب (05/0-01/0 گرم در 100 میلی لیتر)، زمان واکنش (270- 240-210-180-150-120-90-60-30 دقیقه) و pH ( 11-9-7-5-4-3-2) بر حذف رنگ راکتیو قرمز 198 مورد بررسی قرار گرفت. غلظت رنگ در نمونه های مختلف با استفاده از اسپکتروفتومتر در طول موج 518 نانومتر اندازه گیری شد. ایزوترم های جذب رنگ راکتیو قرمز 198 بر روی نانو لوله های کربنی چند جداره بر اساس معادلات جذب ایزوترم لانگمویر، فروندلیچ و بت تعیین شدند. یافته ها: حداکثر ظرفیت جذب سطحی رنگ راکتیو قرمز 198 بر روی نانو لوله های کربنی چندجداره معادل 1000 میلی گرم حاصل شد. از طرفی نتایج مطالعه نشان داد که بیشترین حذف رنگ با غلظت اولیه 50 میلی گرم بر لیتر در 4= pH معادل 4/74 درصد بوده (mg/g 02/372) و در عین حال مشخص گردید زمان مناسب جهت جذب رنگ 3 ساعت می باشد. همچنین داده های حاصل از مطالعه حاضر با ایزوترم جذب لانگمویر (9996/0 = R2) تطابق بیشتری را نسبت به ایزوترم های فروندلیچ و بت نشان داد. نتیجه گیری: بر اساس یافته های مطالعه حاضر مشخص گردید که نانو لوله های کربنی چندجداره از توانایی مطلوبی در حذف رنگ راکتیو قرمز 198 از محلول های آبی و فاضلابهای صنعت نساجی برخوردار است.

Title: بررسی حذف کروم شش ظرفیتی با استفاده از نانولوله های کربنی تک جداره وچند جداره فعال از محلول های آبی

http://tbj.ssu.ac.ir/article-1-1730-fa.html

چکیده مقدمه: فلزات سنگین از جمله آلاینده‌های مهمی هستند که امروزه غلظت آن‌ها در محیط زیست با گسترش شهرها و توسعه صنایع افزایش یافته است. یکی از مهمترین فلزات سنگین، کروم است و به صورت اکسیدهای سه و شش ظرفیتی در صنایعی مانند آبکاری، شیشه، سرامیک، دباغی و چرم کاربرد دارد. کروم شش ظرفیتی دارای مخاطرات بهداشتی اثبات شده ای مانند سرطانزایی است. هدف از این مطالعه بررسی مقایسه ای کارآیی نانولوله های کربنی تک جداره وچند جداره فعال در حذف کروم شش ظرفیتی از محلول‌های آبی است. روش بررسی: این تحقیق یک مطالعه تجربی است که در آن اثر غلظت اولیه کروم، pH ،جرم جاذب و زمان واکنش بر حذف کروم شش ظرفیتی توسط جاذب نانولوله های کربنی تک جداره وچند جداره فعال مورد بررسی قرار گرفته است. برای تهیه جاذب، نانولوله های کربنی تک جداره وچند جداره فعال از پژوهشگاه صنعت نفت ایران (R.I.P.I) تهیه شد. مطالعه روی نمونه‌های سنتزی حاوی غلظت های 5/0، 1 و 2 میلی گرم در لیتر کروم شش ظرفیتی انجام گرفت. غلظت مقادیر کروم شش ظرفیتی به روش جذب اتمی تعیین گردید. یافته ها: با توجه به نتایج، با افزایش زمان تماس و افزایش غلظت آلاینده، ظرفیت جذب (qe) افزایش و با افزایش pH و افزایش جرم جاذب ظرفیت جذب (qe) کاهش یافته است. داده‌های این تحقیق نشان داد که بهترین ظرفیت جذب (qe)کروم شش ظرفیتی توسط نانولوله های کربنی تک جداره وچند جداره فعال در مدت زمان 120دقیقه با pH مساوی 3 و غلظت الاینده 2 میلی گرم بر لیتر و مقدار جاذب 4/0 گرم بر لیتر به ترتیب برابر با 94/3 و 74/3 میلی گرم برگرم بدست آمد و بر اساس نتایج بدست آمده کارائی نانولوله های کربنی تک جداره بهتر از چند جداره بوده است، با اینحال اختلاف معنی داری بین نتایج حذف این دو نوع جاذب مشاهده نشده است. نتیجه گیری: نانولوله های کربنی تک جداره فعال نسبت به چند جداره فعال جاذبی مؤثرتر در حذف کروم شش ظرفیتی از محلول‌های آبی می باشند.

Title: فناوری نانو و حذف آلودگی‌های آب

http://tbj.ssu.ac.ir/article-1-1735-fa.html

چکیده مقدمه: مطالعات نشان داده ‌است که چنانچه تصفیه‌ی فاضلاب و پساب‌ها بطور مناسب مدیریت شود، بخش قابل ملاحظه‌ای از منابع آب مورد نیاز جوامع در حال توسعه را فراهم می‌کند. با افزایش بی‌رویه غلظت مواد ریزدانه، ترکیبات آلی و معدنی و بخصوص فلزات سنگین در پساب‌ها روش‌های متعارف جوابگوی حذف آلاینده‌ها نبوده و لازم است از فرآیندهای جدید در حذف آنها استفاده شود. با توسعه فناوری‌های نوین از قبیل نانو فناوری، فرآیندهای حذف آلاینده‌ها از آب و پساب‌های صنعتی و کشاورزی بهبود یافته است. این مطالعه مروری بر تحقیقات انجام شده در مورد کاربرد فناوری ‌نانو در حذف آلاینده‌های آب است که در این زمینه از سال 2000 تا 2013 منتشر شده است وبا عبارات کلیدی Application of Nanotechnology ، Nanotechnology and wastewater treatment ، Nanofiltration and wastewater در اینترنت و مجلات معتبر علمی بهداشت محیط جستجو انجام شد. نتیجه‌گیری: اکثر مطالعات نشان داده‌اند، که استفاده از مواد نانو به علت دارا بودن سطح ویژه بسیار زیاد و خواص شیمیایی منحصر بفرد به کاربران این امکان را می‌دهد تا بطور مستقیم یا پس از اصلاح و عاملدارشدن به صورت اختصاصی و با ظرفیت بالاتر آلاینده‌ها را از محیط آبی خارج ‌نمایند. نانو فیلتراسیون، نانو فتوکاتالیست‌ها، نانو ذرات آهن صفر ظرفیتی، نانو ذرات مغناطیسی از جمله روش‌ها و موادی هستند که جهت کنترل و حذف آلاینده‌ها از آب و پساب استفاده شده‌اند. حذف آلاینده‌ها و بازیابی آب خالص با بکاربردن فناوری نانو تاثیر زیادی در کاهش هزینه و زمان در صنعت تصفیه داشته و باعث بهبود منابع آبی در محیط زیست می‌گردد. نانو ذرات مغناطیسی اکسید آهن به علت دارا بودن خاصیت مغناطیسی و امکان جداسازی راحت‌تر از محیط پس از تصفیه، یک روش موثر و کارامد در حذف آلاینده‌ها بشمار می‌رود.
